# A Case of Eosinophilic Granulomatosis With Polyangiitis (EGPA) Peripheral Neuropathy With Positive Anti-Myelin Oligodendrocyte (MOG) Antibodies

**DOI:** 10.7759/cureus.48055

**Published:** 2023-10-31

**Authors:** Kauser Yousuf, Hadiza Ibrahim, Mahfoud Elbashari, Mohamed E Abouelnaga, Amani Alzaabi

**Affiliations:** 1 Internal Medicine, Zayed Military Hospital, Abu Dhabi, ARE; 2 Neurology/Internal Medicine, Zayed Military Hospital, Abu Dhabi, ARE

**Keywords:** systemic autoimmune diseases, antineutrophil cytoplasmic antibody (anca) associated vasculitis (aav), anti-mog antibody, peripheral neuropathy, eosinophilic granulomatosis with polyangiitis (egpa)

## Abstract

Peripheral neuropathy is a common manifestation of Eosinophilic Granulomatosis with Polyangiitis (EGPA), a rare autoimmune disorder caused by eosinophilic infiltration of multiple organs including the nervous system. Recent research has shown an association between myelin oligodendrocyte glycoprotein (MOG) antibodies and various neurologic conditions. We present a unique case of EGPA with positive MOG antibodies in the cerebrospinal fluid (CSF) in a patient presenting with peripheral neuropathy. We also highlight a few diagnostic dilemmas with EGPA and the importance of early diagnosis and appropriate treatment. Clinical, laboratory, radiological, and electrophysiologic findings are discussed.

## Introduction

Peripheral neuropathy has a wide variety of causes including metabolic, autoimmune, paraneoplastic and more. It can be challenging to diagnose the underlying cause. Eosinophilic Granulomatosis with Polyangiitis (EGPA), one of the antineutrophilic cytoplasmic antibody (ANCA) associated vasculitides, is a known cause of peripheral neuropathy. It is a multisystem disorder caused by granulomatous inflammation of small to medium blood vessels with eosinophilia leading to dysfunction of affected organs, most commonly the lungs and the skin; it can also affect the renal, cardiovascular, gastrointestinal, central, and peripheral nervous systems hence a wide variety of clinical presentations are possible [1]. In addition to the result of varying clinical presentations, appropriate diagnosis can be delayed due to its rarity, with a prevalence of 10.7 to 14 per million adults worldwide [2].

EGPA is primarily a clinical diagnosis supported by imaging and histopathological studies [3]. A number of different sets of criteria used to aid in the diagnosis of EGPA have been proposed including the American College of Rheumatology (ACR) criteria [4]. The presence of four or more of the six criteria yielded a sensitivity of 85% and a specificity of 99.7% for the classification of vasculitis as EGPA [4]. A high index of suspicion is important for diagnosis and diagnosis is imperative in order to optimize therapy, prevent life-threatening or debilitating complications, and improve the quality of life of patients.

Another reported cause of peripheral neuropathy is MOGAD which stands for Myelin Oligodendrocyte Glycoprotein Disease. MOGAD is a demyelinating disease primarily of the central nervous system. Of late, it has also been found to affect the peripheral nervous system, as well as having associations with other autoimmune conditions [5].

## Case presentation

A 27-year-old man presented to the emergency department with complaints of severe burning pain in his feet, which initially began in his left foot and proceeded to involve the right foot. He reported swelling, occasional numbness, and inability to move his feet.

He was incidentally found to be hypoxemic with a low oxygen saturation of 88% on room air, he was tachypneic with a respiratory rate of 30 cycles/min and tachycardic with a heart rate of 116 beats/min. He had bilateral coarse inspiratory crepitations, worse over the lower zones, bronchial breath sounds over the right lower and left lower lung zones, consistent with bilateral consolidations, and there were no rhonchi. No jugular venous distension and no pitting edema were observed. A foot examination revealed features consistent with peripheral neuropathy affecting the lower limbs. There was right foot swelling, warmth, and tenderness, pulses bilaterally palpable, asymmetrically diminished power and sensation over both feet in all modalities tested, more marked on the right than left. Muscle power was absent (0/5) globally in his right foot and was weak (3/5) in his left foot. Ankle reflexes were absent and plantar reflexes were mute bilaterally, Upper limbs were spared.

On further questioning, he reported that he had been having worsening shortness of breath for the past month, but that did not bother him as much as the pain. He had a cough with some yellowish sputum with no chest pain. He denied any abdominal pain, skin rashes, no burning, or changes in his urine, and no other complaints. He was diagnosed with bronchial asthma over a month prior to presentation, and prescribed Ventolin^TM^ which he used daily with no improvement, he has a history of recurrent sinusitis and nasal polyps, but no history of heart disease. He denied having ever smoked.

**Figure 1:**
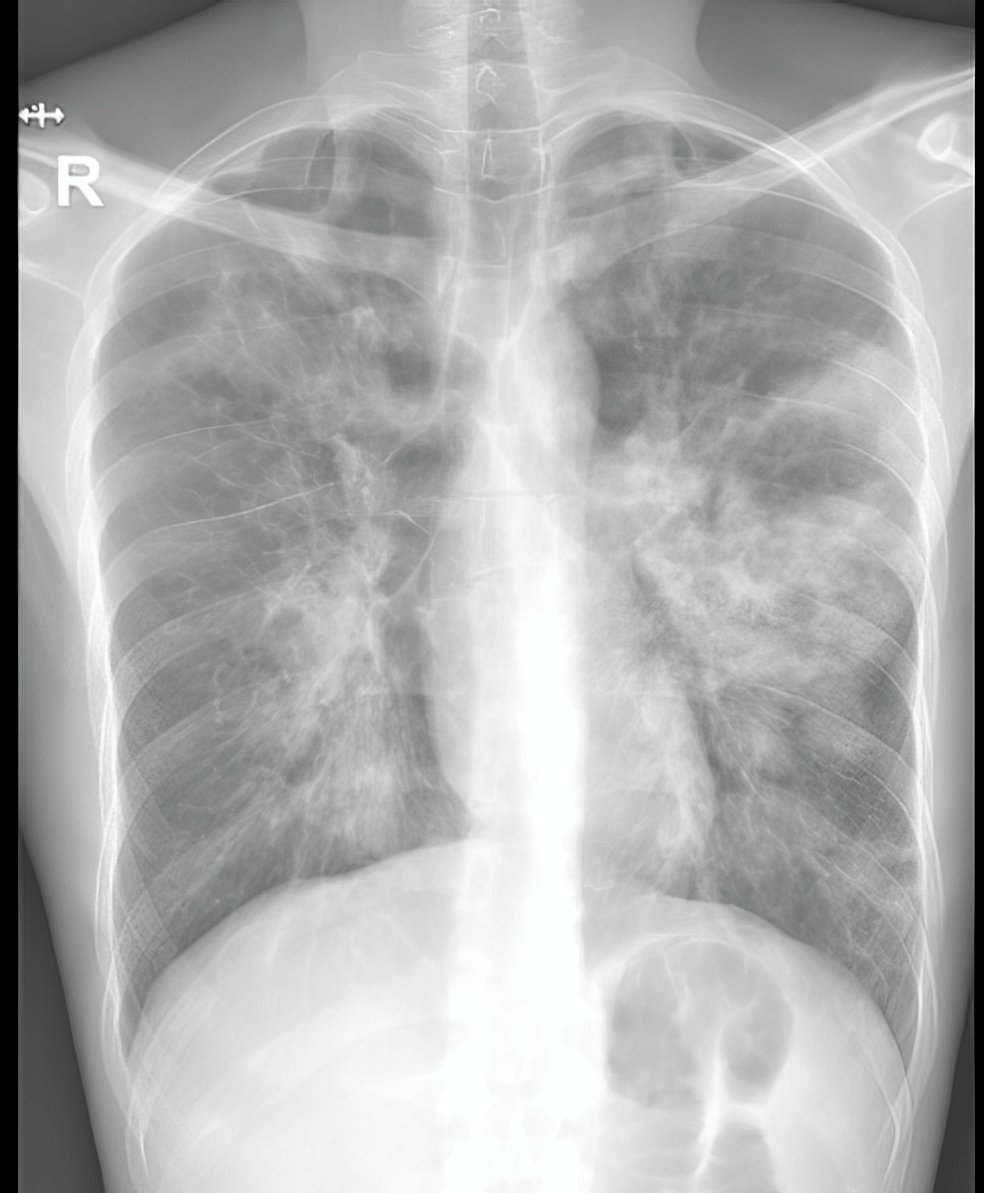
Bilateral infiltrates on initial chest X-ray

He had raised inflammatory markers on blood tests. WBC count of 16000/mm^3^ with a 42.1% eosinophils CRP of 84.9 mg/dL. Electrolytes, liver, and kidney function tests, and urinalysis were normal. He had bilateral opacities on the initial chest X-ray (Figure 1) and a normal transthoracic echocardiogram.

**Figure 2:**
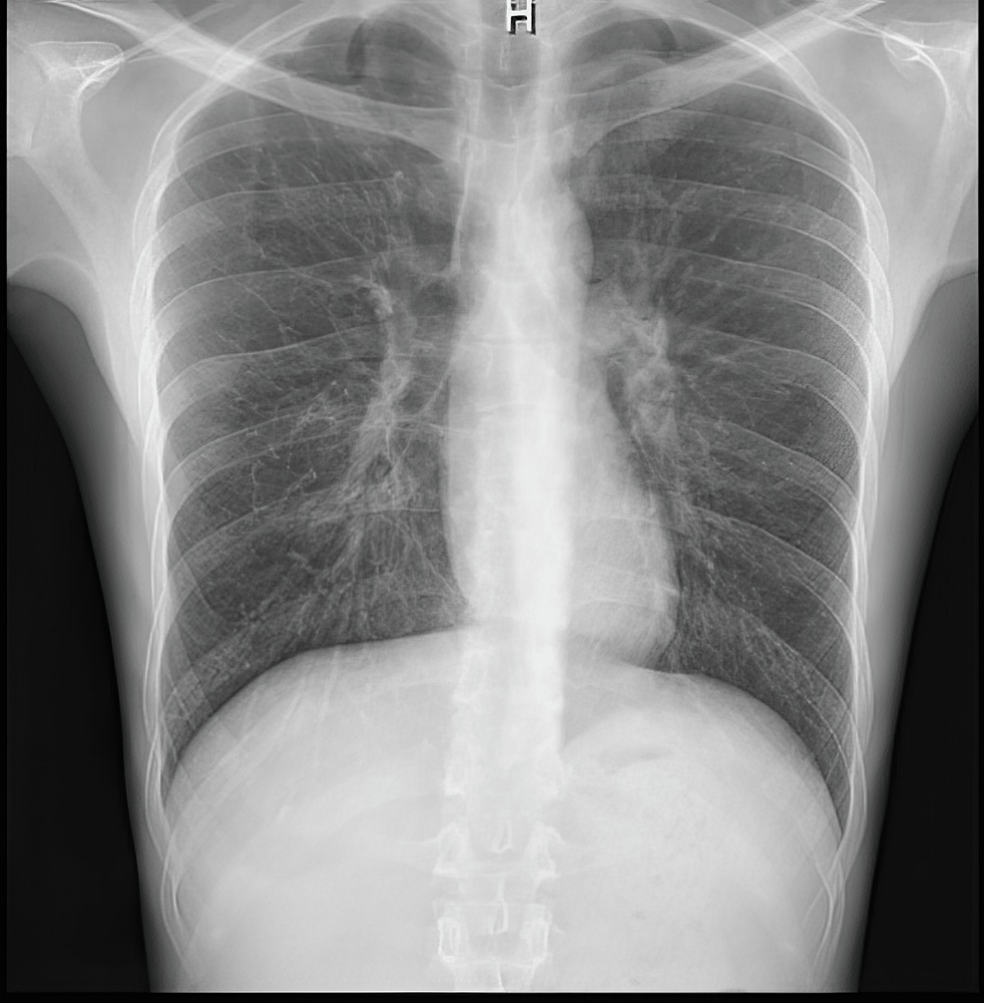
Repeat chest X-ray after a few days into treatment with steroids The X-ray image is showing a complete resolution of lung infiltrates.

He was initially treated for community-acquired pneumonia and started on a seven-day course of ceftriaxone and doxycycline (his blood and sputum cultures never yielded any growth). After a few days into the treatment he was commenced on dexamethasone as the diagnosis of vasculitis became more likely given the multisystem involvement and marked eosinophilia, his inflammatory markers trended down following initiation of steroids, and there was complete resolution of the initial chest X-ray infiltrates (Figure 2).

**Figure 3:**
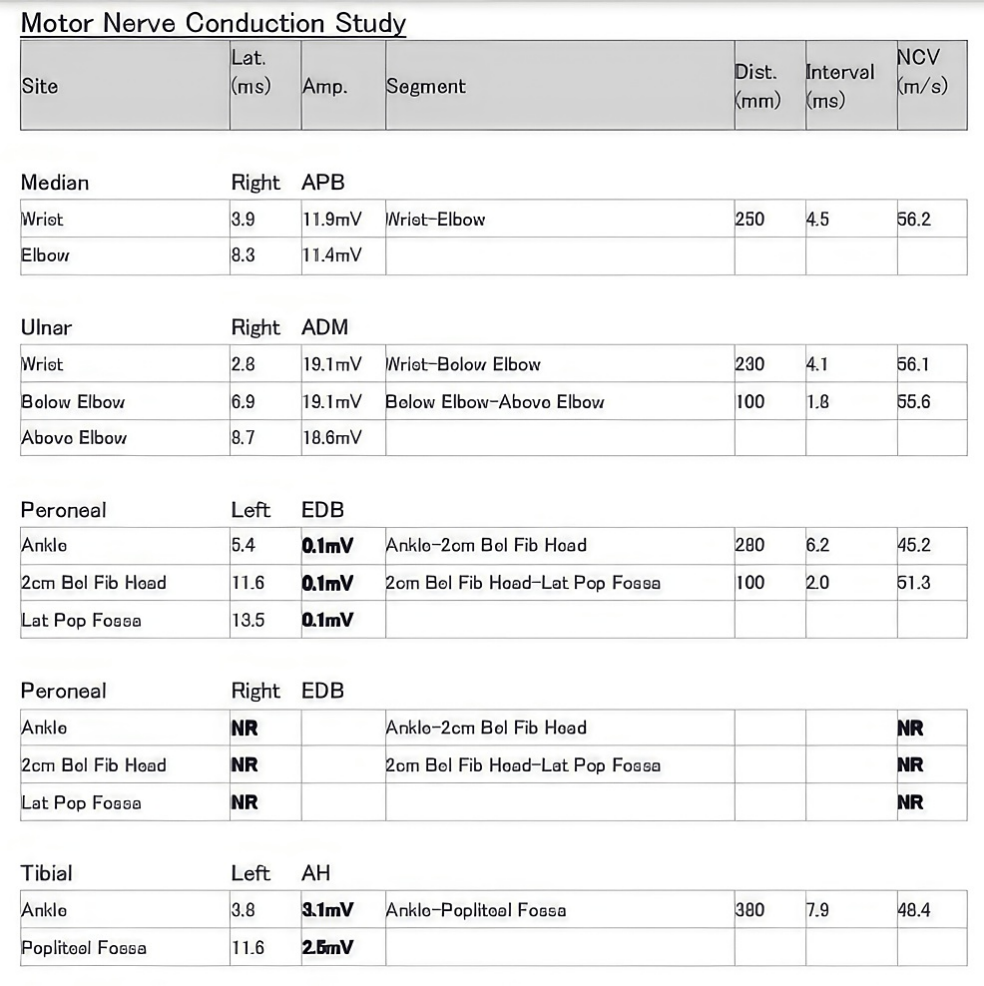
Motor nerve conduction study report The report depicts (i) the absence of elicitable electrical activity in the right peroneal, deep peroneal, and tibial nerves; (ii) normal distal motor latencies and normal conduction velocities with markedly reduced MAP amplitudes from left peroneal, deep peroneal and tibial nerves. Lat: latency; Amp: amplitude, Dist: distance; NCV: nerve conduction velocity; ms: milliseconds; mV: millivolts; mm: millimeters; m/s: meter per second; NR: No response; MAP: muscle action potential.

**Figure 4:**
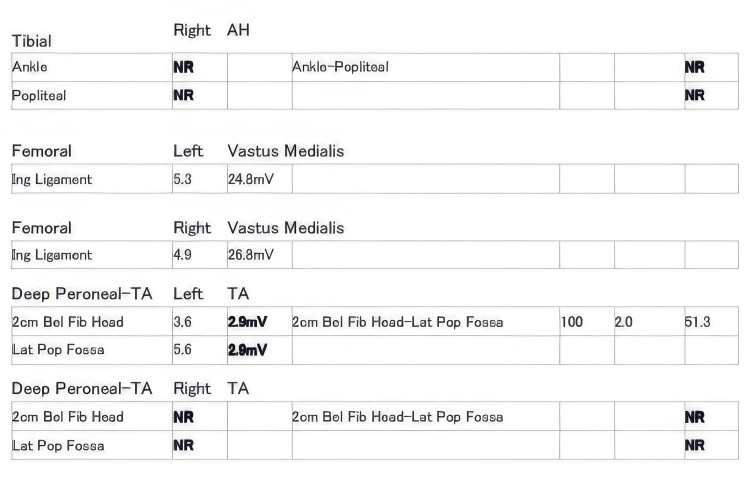
Motor nerve conduction study report The report shows (i) the absence of elicitable electrical activity in the right peroneal, deep peroneal, and tibial nerves and (ii) normal distal motor latencies and normal conduction velocities with markedly reduced MAP amplitudes from left peroneal, deep peroneal and tibial nerves. Lat: latency; Amp: amplitude, Dist: distance; NCV: nerve conduction velocity; ms: milliseconds; mV: millivolts; mm: millimeters; m/s: meter per second; NR: No response; MAP: muscle action potential.

**Figure 5:**
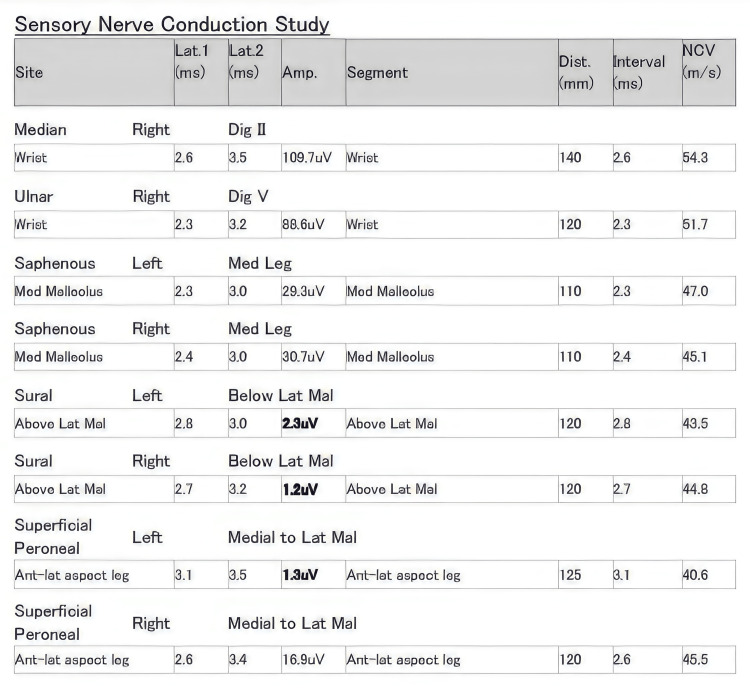
Sensory nerve conduction study The report shows normal sensory latencies and normal conduction velocities with markedly reduced SNAP amplitudes from left superficial peroneal and bilateral sural nerves. Lat: latency; Amp: amplitude, Dist: distance; NCV: nerve conduction velocity; ms: milliseconds; mV: millivolts; mm: millimeters; m/s: meter per second; uV: microvolts; SNAP: sensory nerve action potentials.

**Figure 6:**
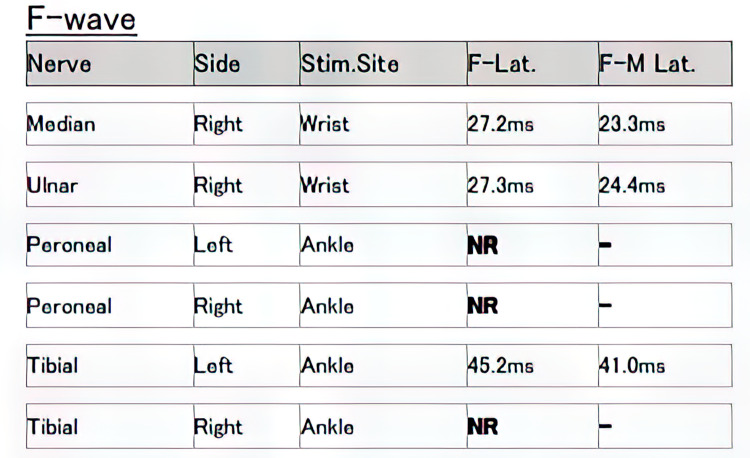
F-wave study showing absent F-waves from right tibial and bilateral peroneal nerves Stim. site: stimulation site; F-Lat: F-wave latency; F-M Lat: F-wave motor wave latency; ms: milliseconds; NR: no response.

**Figure 7:**
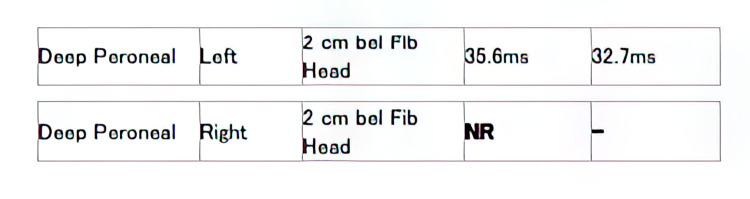
F-wave study showing absent F-waves from right deep peroneal nerve Extension of Figure 6 ms: milliseconds; NR: no response.

His foot pain was persistent throughout admission. He underwent a nerve conduction study which showed features suggestive of asymmetric axonal sensorimotor polyneuropathy involving both distal lower limbs, affecting the right more than the left (Figures 3-7), mononeuritis multiplex. He then underwent a sural nerve biopsy of the most affected leg (right) which came back negative for vasculitis. This caused a bit of quandary with regard to his diagnosis. Serum vasculitic screen however was positive with antineutrophil cytoplasmic antibodies (C-ANCA) titer of 1:20, myeloperoxidase (MPO) antibody 37 RU/ml. Perinuclear anti-neutrophil cytoplasmic antibodies (P-ANCA) and anti-proteinase (PR)3 were negative. He also had a lumbar puncture and CSF studies showed an elevated white cell count, predominant neutrophils, and positive anti-myelin oligodendrocyte antibodies with a titer of 1:40. The diagnosis of MOG antibody disease was entertained and he received a course of intravenous immunoglobulin (IVIG) which did not yield much improvement.

**Figure 8:**
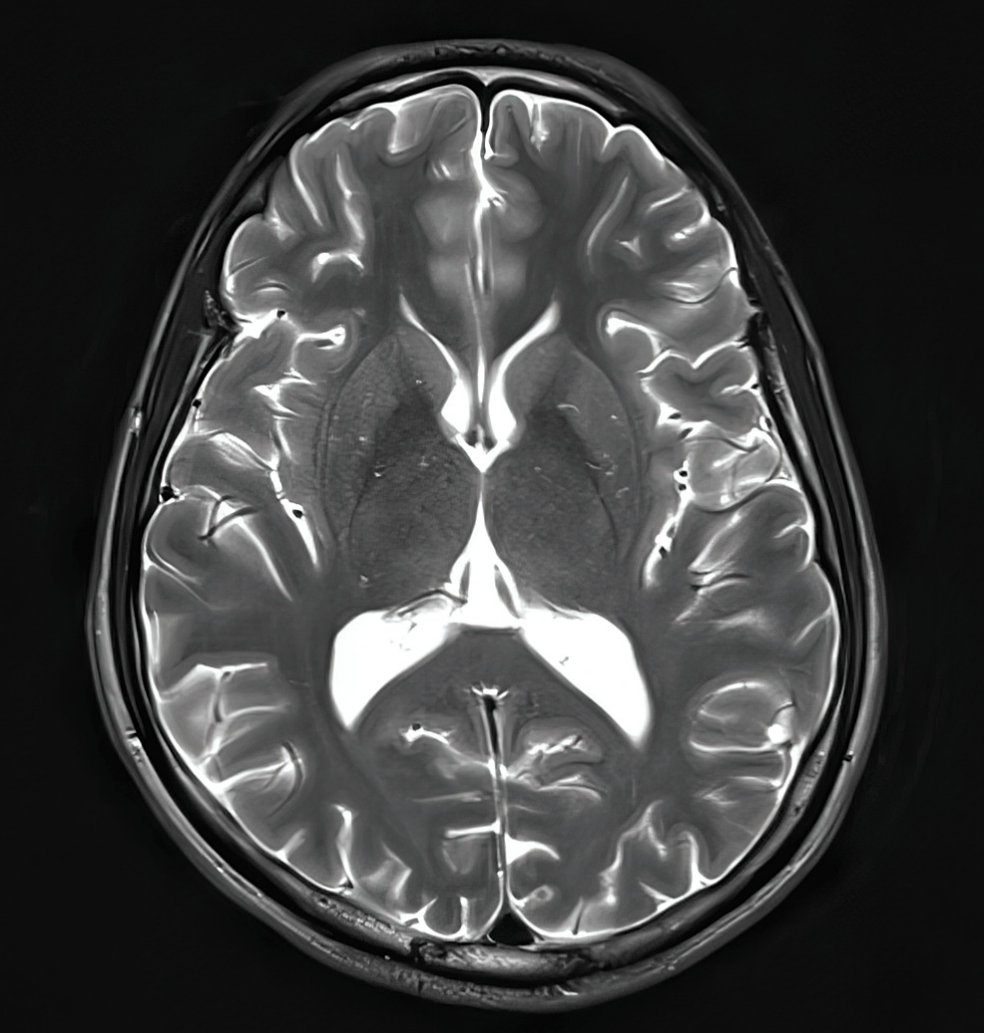
Normal MRI brain, T2 weighted image

**Figure 9:**
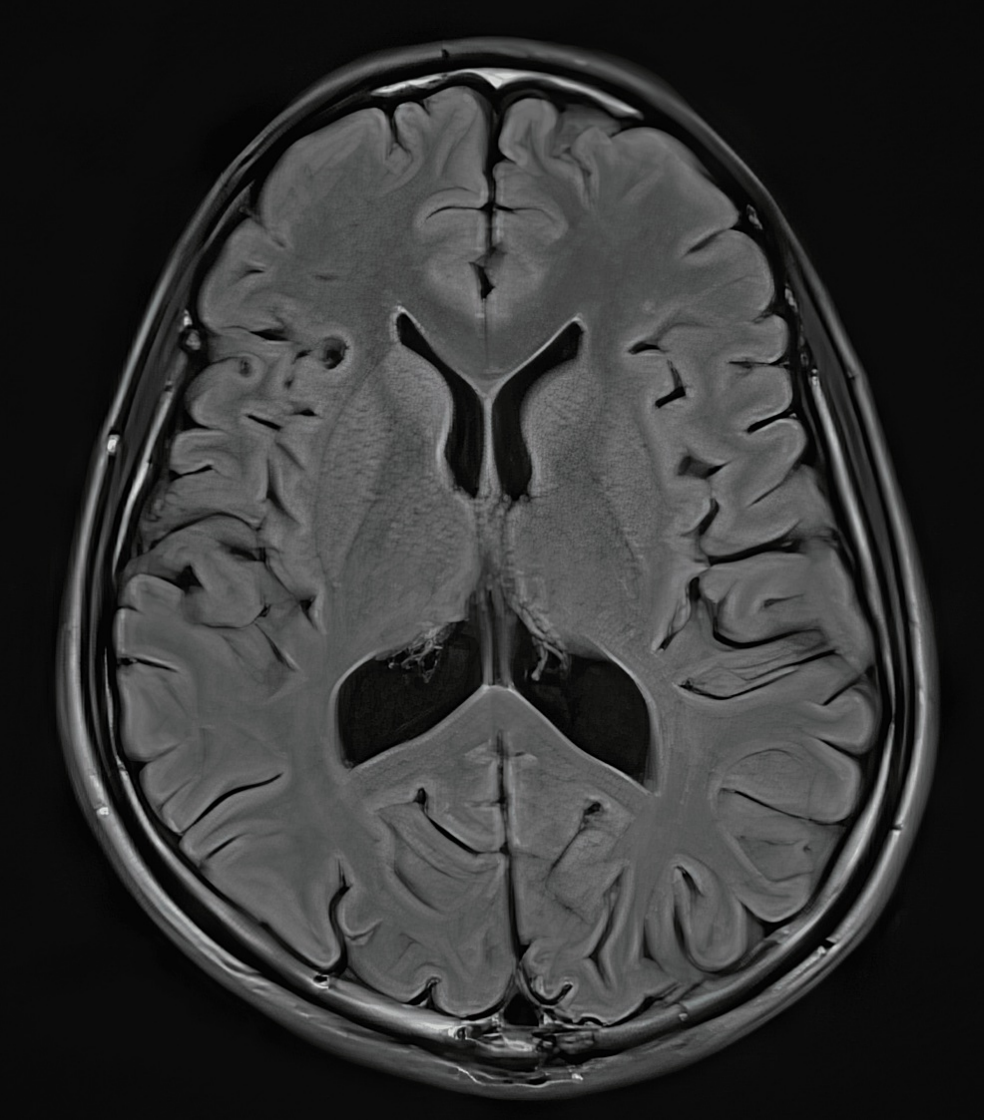
Normal MRI brain, FLAIR sequence FLAIR: Fluid-attenuated inversion recovery.

**Figure 10:**
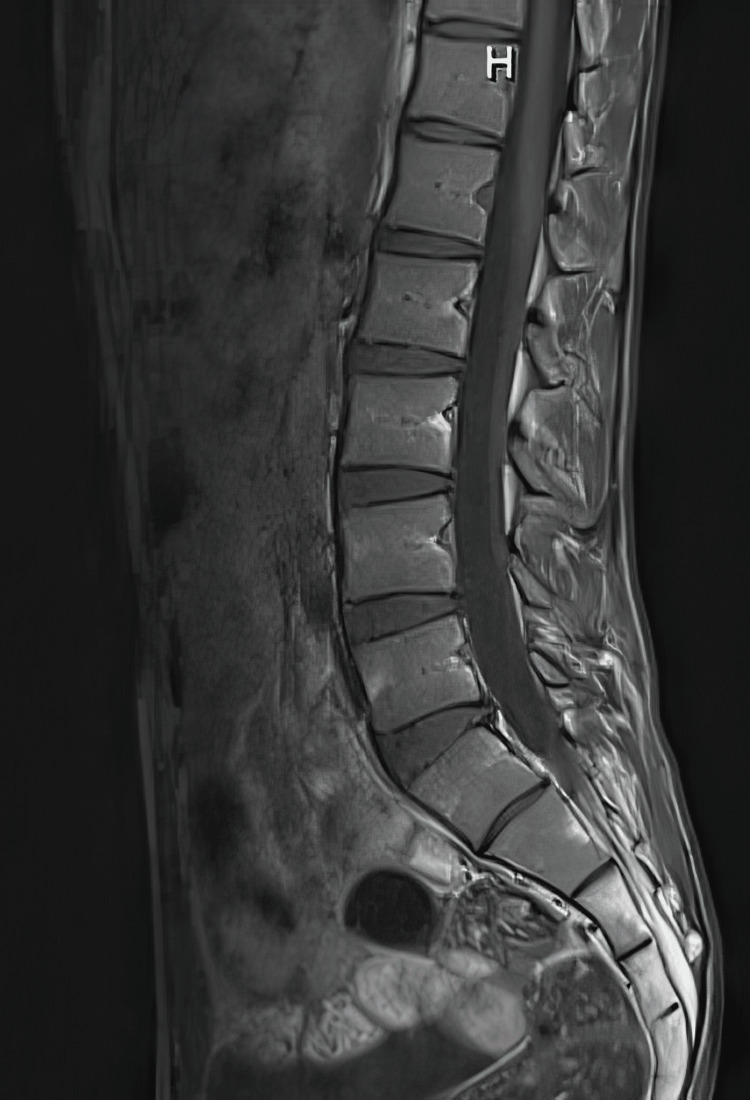
Normal MRI lumbar spine, T1 weighted image

**Figure 11:**
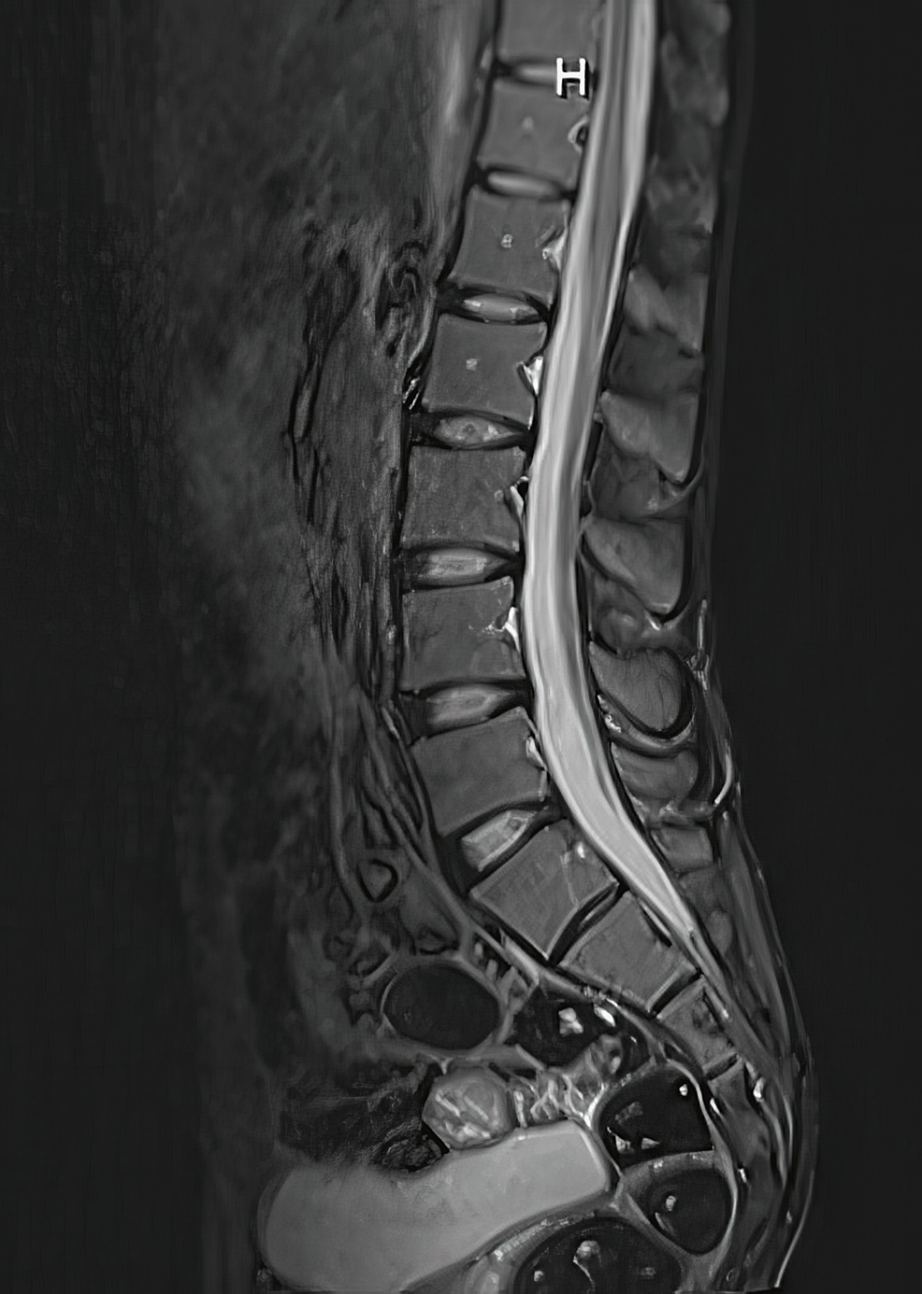
Normal MRI lumbar spine, T2 weighted image

The patient also underwent MRI studies, which were normal (Figures 8-11).

He was diagnosed and treated for vasculitic neuropathy regardless of the biopsy results, as he scored 9 using the ACR criteria as discussed above and also fit the diagnosis of EGPA by Lanham criteria [6]. The anti-MOG antibodies were deemed false positive as the diagnosis of EGPA was much more likely and nerve conduction studies (NCS) were in keeping with an axonal not demyelinating neuropathy. He received five days of IV methylprednisolone 500 mg and later on, changed to oral prednisone 20 mg twice daily. He was commenced on frequent physical therapy and advised to repeat NCS in six months. No lung biopsy was done as his chest findings and symptoms resolved only days after his steroids. He still had persistent foot pain and weakness on steroids, only slightly improved. He ended up frequenting clinics/emergency rooms for pain relief over months. Rituximab was started to arrest disease progression and taper down steroids. Fortunately, his pain resolved and finally, his weakness began improving.

## Discussion

While peripheral neuropathy is a common presentation of antineutrophil cytoplasmic antibodies (ANCA) associated vasculitides with a prevalence >50% in EGPA patients [7], EGPA can be difficult to diagnose. The patchy nature of nerve infiltration often renders biopsy ineffectual with only around 50% of nerve biopsies showing histologic evidence of vasculitis [8]. Biopsy of other affected organs should be considered when possible. A conduction study is helpful to support diagnosis and should show axonal disease.

It is of utmost importance to remember that EGPA is primarily a clinical diagnosis; appropriate treatment should not be delayed in patients who have a very high pretest probability of having the disease as that can come at the cost of significant morbidity to patients.

A high index of suspicion should be maintained to allow for prompt diagnosis and appropriate therapy in this rare condition. Failure to achieve significant improvement with systemic glucocorticoids should prompt the early introduction of rituximab or cyclophosphamide, especially in severe disease [9].

Supportive care in the form of analgesia and physical and occupational therapy should be provided and patients should have regular follow up and appropriate escalation of treatment should be considered early if improvement is poor [10]. Overall, the prognosis of EGPA treated appropriately is good [10]. Patient counseling is also a crucial part of management as well as psychotherapy as with any other chronic diseases. Patients with vasculitis that is initially localized to the peripheral nervous system should be monitored closely as some of these patients may eventually develop vasculitis in other organs.

In our patient, the MOG antibodies found to be positive added to the dilemma of reaching a diagnosis. MOG antibodies in the CSF were found with a titer of 1:40. Even though there were no CNS manifestations of MOGAD, there have been several case reports of MOGAD associated with peripheral neuropathy [5] and Giant Cell Arteritis (GCA), another medium vessel vasculitis which is why it was part of the differential diagnosis. Additionally, MOGAD has been associated with autoimmune conditions like Systemic Lupus Erythematosus (SLE) and Neuromyelitis Optica spectrum disorder (NMOSD) [11] which opened up further discussions. It is important to note however, there are reports of the possibility of a false positive test for MOG-IgG due to cross-reactivity with other autoantibodies [12]. It is unknown to us whether MOGAD was contributing to the patient’s neuropathy or simply a false positive test.

## Conclusions

This case reminds us that EGPA is a clinical diagnosis and biopsy is only an adjunct to diagnosis. There should be appropriate escalation of therapy if needed in patients with EGPA as the most likely diagnosis in order to limit patient morbidity. It also raises the question of the significance of MOG-antibodies in such patients. Is it an innocent bystander (i.e., false positive) or does it have an association with disease severity and/or prognosis? The significance of anti-MOG antibodies in EGPA remains an area of ongoing research and debate.
